# A survey of subjective constipation in Parkinson’s disease patients in shanghai and literature review

**DOI:** 10.1186/s12883-018-1034-3

**Published:** 2018-03-15

**Authors:** Jing Gan, Ying Wan, Junjie Shi, Mingzhu Zhou, Zhiyin Lou, Zhenguo Liu

**Affiliations:** Department of Neurology, Xinhua Hospital Shanghai JiaoTong University, School of Medicine, 1665 Kongjiang Road, Shanghai, 20092 China

**Keywords:** Parkinson’s disease, Constipation, Pre-motor symptoms, ROME III criteria

## Abstract

**Background:**

Constipation is one of the most frequent non-motor symptoms (NMS) in Parkinson’s disease (PD) and the prevalence of constipation in PD patients varies among different studies. We designed this study to survey the prevalence and clinical characteristics of subjective constipation and the appearance chronology between the emergence of constipation and onset of motor symptoms in PD patients from Shanghai, China.

**Methods:**

268 PD patients were continuously recruited into this study. Parkinson’s related clinical information of the participants was collected. A spectrum of motor and nonmotor features was assessed with scales and questionnaires. Subjective constipation was defined by ROME III criteria.

**Results:**

54.10% PD patients suffer from constipation. Among them, there was 47.59% having constipation before onset of motor symptoms. Compared with patients without constipation, patients with constipation reported lower daily water intake and less exercise, and were dominated by bradykinetic-rigid motor phenotype at onset and were prone to have anxiety, depression and insomnia. The time span between constipation and the onset of motor symptoms was (6.62 ± 9.32) years. Constipation occurred more frequently between 2 and 10 years before onset of motor symptoms. Patients suffering with constipation were then divided into two groups according to the time sequence of constipation and motor onset: ‘constipation pre-motor sign’ group and ‘constipation post-motor sign’ group. Total timespan from earliest initial symptoms to present was similar. Compared with ‘constipation post-motor sign’ group, the patients in ‘constipation pre-motor sign’ group experienced an older motor symptoms onset age, less serious motor symptoms, more serious constipation and less daily levodopa dosage.

**Conclusions:**

Our results supported that constipation could be a pre-motor symptom of PD. Different clinical characteristics were found in different constipation-loading time relative to motor symptoms. Research of constipation may be useful to better understand the early stages of PD and assessment of constipation with validated criteria may have utility as a risk factor for predicting PD in the prodromal phase.

## Background

Parkinson’s disease (PD) is a disabling disorder with progressive degeneration of the nigrostriatal pathway that classically impairs motor skills. In the last 15 years, the non-motor symptoms (NMS) of PD became the focus of clinical and scientific interest. Constipation is one of the most frequent and well-known NMS in PD patients [1]. A wide spectrum of prevalence of constipation in Parkinson’s patients has been reported, ranging from 7% to 71% among different studies [2–4]. Constipation appears through all stages of Parkinson’s disease and increases with advancing disease progression [1, 5]. Its impact on the quality of life is no less than motor symptoms. However, there was no suggested or recommended questionnaire/scale for PD constipation and the criteria or definitions of constipation in different studies were heterogeneous [1]. This might contribute to the variation of constipation prevalence, which is not helpful to characterize constipation.

Moreover, constipation is supposed as an early, pre-motor manifestation of PD. Recently, one systematic review and meta-analysis proved that people with constipation have a higher risk of developing PD compared with those without. Constipation may precede the onset of Parkinson’s cardinal motor symptoms by decade [6]. Several reports demonstrated constipation represents a risk factor for PD (determining a relative risk versus control ranging between 2 and 2.5) [2, 6, 7]. As a premotor symptom, the prevalence of constipation and the time interval between the occurrence of constipation and the motor symptom onset in Chinese Parkinson’s patients has not been reported. Therefore, we conducted this cross-sectional investigation in Chinese Parkinson’s patients in Shanghai to clarify the prevalence and clinical characteristics of subjective constipation and to evaluate the chronology of motor symptoms and constipation. Meanwhile, related literature would be reviewed to better understand the variation of constipation and its clinical characteristics between Western population and Asians.

## Methods

### Participants

All PD patients recruited from the Movement Disorders Unit of Xinhua Hospital were eligible to be enrolled into this study from Dec 2013 to Dec 2015. All participants were diagnosed by specialists according to the United Kingdom Parkinson’s Disease Society Brain Bank clinical diagnostic Criteria [8]. Patients with cancer, no neurological handicap, severe general medical limitations, systemic and gastrointestinal disorders (except constipation), prior gastro-intestinal surgery or any other identifiable cause of constipation were excluded from the study. Patients with significant cognitive impairment or use of anticholinergic medicament were also excluded. We interviewed all the patients in person through a face-to-face way and collected the socio-demographic and clinical data at the same time.

This study was approved by the Research Ethics Committee of Xinhua Hospital, Shanghai JiaoTong University, China and the patients provided written informed consent.

### Clinical assessment

For all the patients, socio-demographic and clinical data were collected. In addition, motor and non-motor symptoms were evaluated with the Unified Parkinson’s Disease Rating Scale (UPDRS), Heohn-Yahr (H-Y) stage, Non-Motor Symptoms Questionnaire (NMSQuest), Mini-Status Examination (MMSE), Hamilton Anxiety Scale (HAMA), Hamilton Depression Scale (HAMD), Parkinson’s Disease Sleep Scale (PDSS) and Parkinson’s Disease Questionnaire-39 items (PDQ-39).

Constipation was defined through the ROME III functional constipation criteria [9]. ROME III functional constipation criteria define constipation as the presence of two or more symptoms (straining for defecation, lumpy/hard stools, sensation of incomplete evacuation, sensation of anorectal obstruction, manual maneuvers and less than 3 bowel movements per week) at least 25% of time for more than 3 months with onset at least 6 months. The Cleveland Constipation scoring System (CCS) [10] and Patient Assessment of Constipation Quality of Life scale (PAC-QoL) were also used. The CCS allows graduation of symptoms from mild to severe [10, 11]. In our study, CCS score less than 15 was considered as mild constipation and CCS score equal or greater than 15 was defined as severe constipation.

Among these data, age at motor symptom onset which was defined self-perceived (subjective) motor signs, motor subtype predominance was retrospective. If the patients have constipation, they were asked the presence of constipation as well as the time when they had been first noticed by patients relative to onset of motor symptoms. All examinations were performed in the “on” state, except UPDRS III, which was assessed after absence of anti-parkinsonian medication overnight.

### Statistics

Data are presented as means±standard deviation (SD) or percentage. Independent sample t-tests, non-parametric test and Variance test were used. Spearman correlation analysis was also used. Statistical analysis was performed using the Statistical Package for the Social Sciences 18.0 (SPSS, Chicago, IL, USA), and *p* <  0.05 was considered to be significant.

## Results

### Demographic characteristics

A total of 268 idiopathic consecutive PD patients were included. There were 149 male patients (55.60%) and 119 female patients (44.40%). The mean age was 68.92 ± 9.04 years old (range: 34–90 years) with a mean disease duration of 5.75 ± 4.84 years (range: 0.40–22 years).

### Point prevalence and clinical features of PD patients with subjective constipation

Among 268 PD patients interviewed, 145 (54.10%) patients fulfilled the criteria for constipation. PD patients were then divided into two groups: the group of patients with constipation (constipation group) and the group of patients without constipation (non-constipation group). Socio-demographic and clinical characteristics were compared between the two groups (Table 1). There was no significant difference in age, gender ratio between the two groups. In life style, the proportion of “moderate exercise” [12] and “moderate drinking” [13] in the constipation group was significantly lower than that in non-constipation group (23.45% versus 34.96% and 56.55% versus 69.11%, respectively). In terms of clinical characteristics, there was no difference in age of motor symptoms onset, disease duration (equivalent to Duration of motor signs), UPDRS III score and H-Y stage between the two groups. Both the two groups presented with the tremor dominant motor phenotype, however, the percentage of patients with tremor dominant motor phenotype in the constipation group (59.31%) was less than that in non-constipation group (71.54%). The scores of non-motor syndromes (NMSS), HAMA, HAMD and quality of life (PDQ-39) were much higher in constipation group than those in non-constipation group. The PDSS score was lower obviously in PD constipation group, compared with PD non-constipation group. Both the daily levodopa dosage and LED in constipation group were much higher than those in non-constipation group (581.11 ± 345.05 mg/d vs 467.60 ± 327.54 mg/d and 705.02 ± 417.86 mg/d vs 518.87 ± 378.51 mg/d, respectively). However, there was no significant difference in daily levodopa dosage per body weight between the two groups.

**Table 1:** Comparison of PD patients with/without constipation

Item	PD with constipation	PD without constipation	Test value	*P* value
Number of case	*N* = 145	*N* = 123	/	/
Male/Female^b^	83/62	66/57	χ^2^ = 0.346	0.622
Age (year)^a^	69.49 ± 10.06	67.69 ± 9.80	*t* = − 1.472	0.142
BMI^a^	22.69 ± 3.03	22.63 ± 2.77	*t* = 0.157	0.876
Life Style				
Moderate exercise^b^	33(22.76%)	43(34.96%)	χ^2^ = 4.876	*< 0.05*
Moderate drinking^b^	80(55.17%)	85(69.11%)	χ^2^ = 5.460	*< 0.05*
Smoking^b^	13(8.97%)	8(6.50%)	χ^2^ = 0.558	> 0.05
Parkinsonism clinical characteristic				
Age of motor onset (year)^a^	63.75 ± 9.51	62.67 ± 10.45	*t* = −0.873	0.383
Phenotype of onset^b^				
tremor-dominant	86(59.31%)	88(71.54%)	χ^2^ = 5.109	*< 0.05*
Bradykinetic-rigid dominant	54(37.24%)	30(24.39%)		
others	5(3.45%)	5(4.07%)		
Disease duration (year)^a^	6.29 ± 4.95	5.13 ± 4.65	*t* = −1.946	0.053
Dose of levodopa (mg/d)^a^	581.11 ± 345.05	467.60 ± 327.54	*t* = −2.589	*0.010*
LED (mg/d)^a^	705.02 ± 417.86	518.87 ± 378.51	*t* = −3.576	*0.000*
Daily levodopa/kg (mg/kg)^a^	9.11 ± 6.75	7.94 ± 6.00	*t* = −1.399	0.163
UPDRS score^a^	43.91 ± 25.74	35.86 ± 21.14	*t* = −2.783	*0.006*
UPDRSIII score^a^	25.82 ± 15.45	22.61 ± 14.81	*t* = −1.708	0.089
H-Y^a^	2.29 ± 0.87	2.16 ± 1.41	*t* = −0.900	0.369
NMSS^a^	52.77 ± 42.32	31.70 ± 28.18	*t* = − 4.655	*0.000*
MMSE^a^	26.38 ± 3.86	26.65 ± 3.97	*t* = −0.553	0.581
PDSS^a^	111.48 ± 26.15	119.17 ± 25.60	*t* = −2.222	*0.027*
HAMA^a^	11.89 ± 8.80	9.42 ± 7.60	*t* = 2.782	*0.006*
HAMD^a^	15.37 ± 11.38	12.05 ± 10.03	*t* = 2.361	*0.019*
PDQ-39^a^	35.82 ± 28.23	25.50 ± 24.18	*t* = −3.089	*0.002*
CCS	13.16 ± 5.65	–	–	**–**

Moderate exercise [12]: For adult, daily total physical activity is equivalent to at least 6000 steps accumulatively. Moderate drinking [13]: 1.5-2 L/day

LED: Levodopa equivalent doses were calculated as 100 mg L-dopa = 10 mg bromocriptine = 50 mg piribedil = 1 mg prampexole [5]

Statistial significance (p< 0.05) in italics

^a^statistic with t-test

^b^statistic with χ2 test

In the aspect of the severity of constipation, in the constipation group, CCS score was in positive association with constipation duration (*r* = 0.303, *p* = 0.000), daily levodopa dosage per body weight (*r* = 0.169, *p* = 0.021), NMSS (*r* = 0.224, *p* = 0.011), PAC-QoL (*r* = 0.618, *p* = 0.000) and PDQ39 (*r* = 0.231, *p* = 0.010).

### Clinical features of PD patients with constipation preceding motor symptoms

Our study investigated the onset of constipation retrospectively. Among 145 PD patients with constipation, there were 69 patients (47.59%) who have constipation before PD motor symptoms onset (including tremor, slow movement or rigidity etc). According to the time sequence of constipation and motor symptoms onset, the patients with constipation were also divided into two groups: the group of patients with the premotor symptom of constipation (‘constipation premotor sign’ group) and the group of patients with constipation after the motor symptom onset (‘constipation post-motor sign’ group). The demographic and clinical characteristics of these two groups were shown in Table 2. Socio-demographic and clinical characteristics were compared between the two groups. The motor symptoms onset age and PD diagnosis age were much older in the ‘constipation premotor sign’ group (66.83 ± 9.28 and 67.02 ± 8.94 years, respectively), compared with ‘constipation post-motor signs’ group (61.20 ± 8.93 and 62.88 ± 9.56 years, respectively), (*p* = 0.000 and 0.020, respectively). No statistically significant difference was found in the time interval from motor symptoms onset to PD diagnosis between the two groups. Correspondingly, the time from motor symptoms onset to PD diagnosis was much shorter in the ‘constipation pre-motor signs’ group than that in the other group (3.74 ± 3.96 versus 8.50 ± 4.66, respectively, p = 0.000).

**Table 2:** Comparison of clinical features of PD with constipation pre-motor signs and constipation post-motor signs

Item	constipation pre-motor signs	constipation post-motor signs	Test value	*P* value
Number of case	*N* = 69	*N* = 76	/	/
Male/Female^b^	33/36	47/29	χ^2^ = 2.873	> 0.05
Age (year)^a^	70.45 ± 8.41	68.66 ± 11.30	*t* = 1.062	0.290
BMI^a^	22.91 ± 3.17	22.49 ± 2.92	*t* = 0.704	0.483
Moderate exercise^b^	15(21.74%)	18(23.68%)	χ^2^ = 0.078	> 0.05
Moderate drinking^b^	34(49.28%)	46(60.53%)	χ^2^ = 1.851	> 0.05
Smoking^b^	7(10.14%)	6(7.89%)	χ^2^ = 0.224	> 0.05
Age of motor onset (year)^a^	66.83 ± 9.28	61.20 ± 8.93	*t* = 3.640	*0.000*
Age of diagnosis PD (year)^a^	67.02 ± 8.94	62.88 ± 9.56	*t* = 2.353	*0.020*
Motor onset to diagnosis (year)^a^	1.01 ± 0.97	1.51 ± 1.71	*t* = −1.955	0.053
Phenotype of onset^b^				
tremor-dominant	43(62.32%)	43(56.58%)	χ^2^ = 3.322	> 0.05
Bradykinetic-rigid dominant	22(31.88%)	32(42.11%)		
others	4(5.80%)	1(1.32%)		
Duration of motor signs (year)^a^	3.74 ± 3.96	8.50 ± 4.66	*t* = −6.507	*0.000*
Duration of constipation^a^	10.33 ± 10.13	4.48 ± 3.57	*t* = 4.521	*0.000*
Dose of levodopa (mg/d)^a^	467.89 ± 321.25	671.06 ± 338.72	*t* = −3.488	*0.001*
LED (mg/d)^a^	576.40 ± 368.61	807.22 ± 428.69	*t* = −3.254	*0.001*
Daily levodopa/kg (mg/kg)^a^	6.86 ± 6.05	11.15 ± 6.74	*t* = −4.019	*0.000*
UPDRS score^a^	37.85 ± 24.96	49.55 ± 25.33	*t* = −2.759	*0.007*
UPDRSIII score^a^	22.85 ± 14.72	28.58 ± 15.69	*t* = −2.229	*0.027*
H-Y^c^	2.02 ± 0.78	2.54 ± 0.87	*t* = 3.714	*0.000*
NMSS^a^	46.68 ± 39.06	58.85 ± 44.82	*t* = −1.663	0.099
MMSE^a^	26.68 ± 3.26	26.09 ± 4.37	*t* = −0.858	0.393
PDSS^a^	113.11 ± 26.27	110.08 ± 26.17	*t* = −0.618	0.538
HAMA^a^	12.10 ± 9.45	11.69 ± 9.47	*t* = −0.243	0.809
HAMD^a^	14.87 ± 11.57	15.84 ± 11.26	*t* = 0.477	0.634
PDQ-39^a^	31.79 ± 26.66	39.67 ± 29.34	*t* = −1.593	0.114
Mild constipation^b^	30(43.48%)	48(63.16%)	χ2 = 6.161	*< 0.05*
Severe constipation^b^	38(55.07%)	26(34.21%)		
CSS^a^	14.35 ± 5.43	12.00 ± 5.66	*t* = 2.490	*0.014*
PAC-QOL^a^	71.80 ± 29.36	70.26 ± 25.20	*t* = 0.287	0.775

Moderate exercise: For adult, daily total physical activity is equivalent to at least 6000 steps accumulatively. Moderate drinking: 1.5-2 L/day

LED: Levodopa equivalent doses were calculated as 100 mg L-dopa = 10 mg bromocriptine = 50 mg piribedil = 1 mg prampexole [5]

Statistial significance (p< 0.05) in italics

^a^statistic with t-test

^b^statistic with χ^2^ test

With regard to PD motor symptoms and anti-parkinsonian treatment, compared with the ‘constipation post-motor signs’ group’, the ‘constipation pre-motor signs’ group experienced a much lower UPDRS total score, UPDRS III score and H-Y stage and took less daily levodopa dosage and had lower LED and daily levodopa dosage per body weight. In aspect of features of constipation, the ‘constipation pre-motor signs’ group had much longer constipation duration and higher percentage of patients with severe constipation than patients in ‘constipation post-motor signs’ group. Equally, CSS score was higher in ‘constipation pre-motor signs’ group (14.35 ± 5.43) than that in ‘constipation post-motor signs’ group (12.00 ± 5.66) (*p* = 0.014). There was no difference in gender, age, life style or other clinical characteristics between these two groups (Table 2).

**Fig. 1:**
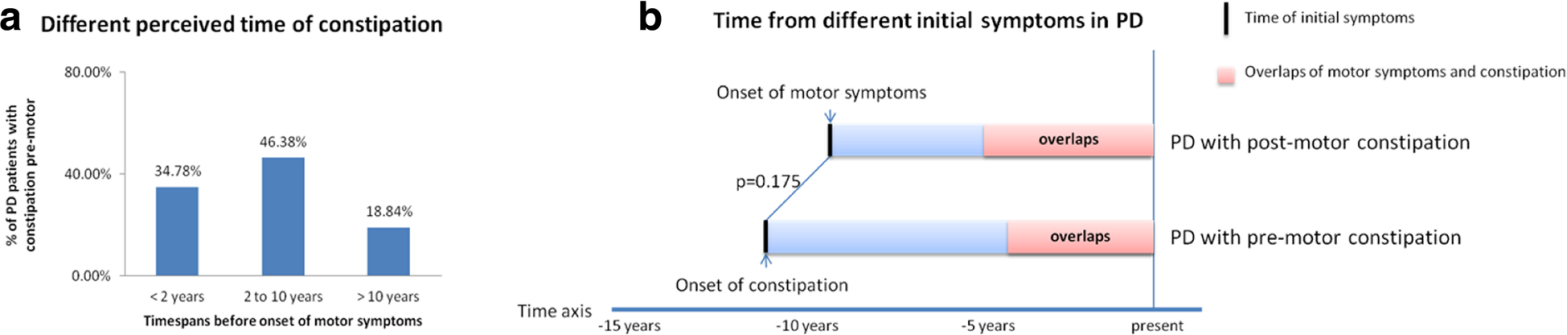
**a** Proportion of PD patients in different perceived time of constipation before onset of motor symptoms. 34.78% patients PD perceived constipation less than 2 years before onset of parkinsonian motor symptoms. 46.38% PD patients perceived constipation between 2 and 10 years before onset od motor symptoms. The rest 18.84% patients occured constipation more than 10 years before motor symptoms. **b** Time from different initial symptoms in PD. There was no difference between time from initial motor symptoms in ‘PD with post-motor constipation’ group and time from intial conspation in ‘PD with pre-motor constiaption’ group. (8.50 ± 4.66 versus 10.33 ± 10.13 years, *t* = 1.367, *p* = 0.175)

The mean time interval from the appearance of constipation to motor symptoms onset in the ‘pre-motor constipation’ group was (6.62 ± 9.32) years. There was no difference in the lead-time in tremor-dominant patients compared to those with non-tremor-dominance (7.23 ± 10.80 versus 5.62 ± 6.19 years, *t* = 0.692, *p* = 0.491). Constipation occur more frequently between 2 and 10 years before the motor symptoms onset (46.38%) (Fig. 1a). If we regard constipation as one initial symptoms of PD, disease duration in ‘constipation pre-motor signs’ group would be changed with data of ‘duration of constipation’. There was no difference between duration of constipation in ‘constipation pre-motor signs’ group and duration of motor signs in ‘constipation post-motor signs’ group (*t* = 1.367, *p* = 0.175) (Fig. 1b).

### Comparison with previous studies

In this study, we selected ROME III criteria for constipation, which was developed and widely accepted by gastroenterologist [1, 14]. According to the review by Knudsen et al. [1] in 2017, there was lack of the summary of this assessment. Here we resumed the prevalence of subjective constipation in previous studies [15–18] which were assessed by ROME III criteria. We reviewed the results of four researches (two populations) with prevalence of PD constipation 50.88% and 59% respectively (Table 3). In fact, the first three studies were conducted by the same research group. Therefore, the pooled estimate in Italy and UK study population showed in Table 3 was 52.06%, which was close to our findings.

**Table 3:** Review and comparison with previous studies

Review of prevalence of constipation in PD according ROME III criteria								
Study		Study population		Sample size (PD patients/Controls)	N of PD patients with constipation	Prevalence	Pooled prevalence	
Barichella et al. (2013) [15]		Italy		208/0	124	59.6%	50.88%	
Cassani et al. (2015) [16]				102/50	58	57%		
Barichella et al. (2016) [17]				600/600	281	46.83%		
Kaye J et al. (2006) [18]		UK		156/148	92	59%	–	
The present study		China		268/0	145	54.10%	–	
Review of prevalence and timespans of constipation before onset of motor symptoms								
Study	Study population	Sample size (PD/Control)	Special inclusion criteria for PD	Criteria for constipation	Constipation prevalence at the time of evaluation	Prevalence of pre-motor constipation (% of PD subjects)	Timespans before onset of motor signs	
							Years (mean ± SD)	proportion
Pont-Sunyer et al. (2015) [19]	Spain and Austria	109/107	untreated PD patients	Specific questionnaire	38.5%	78%	–	< 2 years 21% 2–10 years 15% > 10 years 63%
Cersosimo et al. (2013) [20]	Argentina	129/120	no	Less than three bowel movements per week	53.6%	87%	4 ± 4.6	–
Rodriguez-Violante et al. (2016) [21]	Mexico	500/0	no	NMSS	–	32.8%	9.25 ± 17.89	–
Schrag et al. (2015) [22]	UK	8166/46755	no	Database or use of laxatives	–	28.19%	–	< 2 years 32% 2–5 years 25% 5–10 years 20%
The present study	China	268/0	no	ROME III	54.10%	47.59%	6.62 ± 9.32	< 2 years 34.78% 2–10 years 46.38% > 10 years 18.84%

Then, we reviewed retrospective studies published since 2010 [19–22]. These studies all emphasized time period when constipation had been first noted in relation to the onset of motor symptoms. As listed in Table 3, the prevalence of pre-motor constipation was range from 28.19%–87%. The lead-time of constipation to motor symptom onset was different from (9.2 ± 17.89 years) in Mexico group, (4 ± 4.6 years) in Argentina group, respectively. The ONSET PD study [19] reported that constipation tended to occur more than 10 years before onset of motor signs. Schrag et al. [22] showed that the incidence of constipation was much higher in those finally developed PD than in control at 10 years before diagnosis of PD.

## Discussion

To our knowledge, there are few reports focused on the frequency of PD constipation and on the chronology of appearance of motor symptoms and constipation in the Chinese population. In our study, 54.10% PD patients were ascertained to have subjective constipation by using the ROME III criteria. As we mentioned above, constipation prevalence in a certain population would be partially determined by the specific constipation definition. Knudsen et al. [1] reviewed no less than 12 different definitions used in current PD researches and resumed the prevalence depending on different criteria employed. In this review, the prevalence of constipation ranges from 8%–70% in PD patients and it would reach a median value of 50% (20–63%) according to the most commonly used definition (< 3 bowel movements per week). The pooled prevalence of subjective constipation in PD patients was between 40% and 50%, which was relatively lower than our result. However, Knudsen et al. found that constipation in these studies was not diagnosed by the ROME III criteria which was widely accepted criteria for constipation [14]. The ROME III criteria for constipation was developed through international consensus and widely accepted in the gastroenterological community. 4 PD related studies defined constipation with ROME III criteria and found the prevalence of constipation was ranging from 46.83% to 59.6% (pooled prevalence, 52.06%). The constipation prevalence in our study was consistent with this pooled estimate. Recently, a colon transit and volume study demonstrated that subjective constipation symptom assessed by questionnaires was considerably less prevalent than objective dysfunction [11]. These results indicated that a validated PD constipation questionnaire was required for constipation investigations.

Then, we analyzed the characteristics of PD patients with or without constipation. Compared to patients without constipation, the parkinsonians with constipation reported lower daily water intake and less exercises. These patients were dominated by bradykinetic-rigid onset phenotype and they were more prone to be accompanied with anxiety, depression and sleep disorders, as well as worse quality of life. There was no significant difference in the severity of motor symptoms between these two groups. Ueki A et al. [23] reported that PD patients showed that intake of water was significantly decreased in PD patients from early life and associated with their constipation. Our result also showed that PD patients with constipation took more anti-parkinsonian medicament than whom without constipation. However there was no difference in the daily levodopa dosage per kg between these two groups. Some researchers found dopaminergic treatment may contribute to constipation, especially dopamine agonists [3, 24]. These findings suggested that a life style of drinking more and exercising more should be recommended to the PD patients with non-tremor-dominant phenotype. Early monitoring and treatment of their anxiety, depression, insomnia was necessary to ameliorate their quality of life.

Next, in PD patients with constipation, we retrospectively investigated the timespan between onset of constipation and onset of motor symptoms to clarify the difference of clinical characteristics between PD patients with the pre-motor sign of constipation and PD patients with the post-motor sign of constipation. Our data showed that there were 47.59% of patients having constipation before motor symptoms onset. According to the previous studies we summed up (Table 3), the prevalence of constipation as a pre-motor sign was ranging from 28.19% to 87% depending on the type of studied population and constipation criteria. Compared with the group of PD patients with constipation post-motor symptoms, the group of PD patients with constipation as a pre-motor sign presented with shorter diagnosis duration and milder motor symptoms, took less daily levodopa dosage and experienced a better quality of life. In the aspect of constipation, the group of PD patients with constipation as a pre-motor sign had a longer duration of constipation and a higher percentage of severe constipation.

In recent years, pre-motor symptoms in PD received increasing attention and became one of PD prodromal phase searching. Constipation, as one of the most important NMS, is widely accepted to precede motor symptoms onset several years (the earliest as 20 or more years) [1, 2, 6, 25]. Here we reviewed retrospective studies that reported constipation prevalence and occurrence before motor symptoms onset. Our present result showed that the lead-time of constipation to motor onset (6.62 ± 9.32) years was different from the recent retrospective study (9.2 ± 17.89 years) in Mexico group and (4 ± 4.6 years) in Argentina group, respectively. Meanwhile, as Fig. 1a showed, we found constipation occurred more frequently between 2 and 10 years before onset of motor symptoms. This was consistent with the result of Schrag et al. [22], but the proportion of constipation was obviously lower in the > 10 years period before motor symptoms onset compared to The ONSET PD study [19] (18.84% and 63%, respectively). We hypothesized that these discrepancies were mainly attributed to the following three reasons. Firstly, of course, different constipation questionnaires were used and sample sizes were various. Secondly, the different populations with demographic difference in eating habits, physical exercises and other factors may exhibit variation. Thirdly, the assessment of chronology in the appearance of symptoms was retrospective and subjective. It is not always reliable. The longer timespan, the less reliable.

Although patients who had pre-motor constipation presented motor signs with a more advanced age than the other, Fig. 1b showed that the total timespan from the earliest initial symptom, either constipation or classic motor symptom, was similar (10.33 ± 10.13 versus 8.50 ± 4.66 years, *p* <  0.05). It might be suggested that constipation was one part of PD or a manifestation of early PD and pre-motor constipation load may be associated to a later motor onset of PD. This could be supported by theory of Braak et al. [26] who supposed that the earliest neuropathological features of PD are found in the enteric autonomic nervous system, Lewy neuritis and Lewy bodies appear in the dorsal nucleus of vagus in the earliest stage of the disease and then extend upward through the brain stem to reach the substantia nigra in the third stage when manifested motor symptoms. Several evidences demonstrated that in most PD patients, their entire gut was compromised by a-SYN pathology [2, 27]. They also hypothesized that some yet undefined toxins break through the mucosal barrier of the intestine and were incorporated into the axon terminal of the vagus nerve and transported in a retrograde manner to the vagus nucleus. Enteric sympathetic denervation of rotenone treated mice was considered as an initial premotor alteration in PD progression [28]. In the study of Rodriguez-Violante et al. [21], constipation, with pain and anxiety were the first pre-motor symptoms to manifest related to motor onset and diagnosis. Therefore, constipation could be the first perceived symptom of PD, but its specificity for future PD is not high [29]. Constipation should combine with other specific non-motor symptoms, such as smell lose, dream-enacting behavior, to increase the ability for discriminating PD from control [19].

Finally, our results did not show the association between motor phenotypes and pre-motor constipation, which was consistent with the study of Rodriguez-Violante [21]. However, ONSET-PD study demonstrated that constipation occurred more frequently in akinetic-rigidity phenotype patients in comparison to the tremor-dominant phenotype [19]. More researches should be conducted to clarify this association. Our study has some limitations. This is a retrospective clinical study in which the information of chronology of constipation and motor symptoms were collected through patients’ recalls. Therefore, recall bias could not be ignored and might partially influence the accuracy of our results. Since this is a single-center retrospective study, the results still requires to be testified by more studies including multicenter clinical studies.

## Conclusion

Our findings provided further evidence supporting that constipation was an important pre-motor symptom of PD and that it frequently preceded the onset of motor symptoms. Our data suggested that constipation was an early manifestation of neurodegenerative process underlying PD and therefore part of the disease itself. In daily clinical practice, constipation remained a subjective complains. Therefore, employing for constipation in PD would be necessary and imperative. We recommended for non-tremor-dominant PD patients to drink more, do exercise more and monitor their mood and sleep disorders in early phase to ameliorate quality of life. Constipation could have the potential sensitivity to be used as clinical predict factor of the prodromal phase of disease.

## Abbreviations

CCS: Cleveland Constipation scoring System
H&Y: Hoehn-Yahr stage
HAMA: Hamilton Anxiety Scale
HAMD: Hamilton Depression Scale
LEDD: Levodopa equivalent daily dose
MMSE: Minimum Mental State Examination
NMS: Non-motor symptom
PAC-QoL: Patient Assessment of Constipation Quality of Life scale
PD: Parkinson’s disease
PDQ-39: Parkinson’s Disease Questionaire-39
PDSS: Parkinson’s Disease Sleep Scale
UPDRS: Unified Parkinson’s disease Rating Scale
